# Complex metacommunity structure for benthic invertebrates in a low‐diversity coastal system

**DOI:** 10.1002/ece3.1767

**Published:** 2015-10-22

**Authors:** Sebastian Valanko, Jani Heino, Mats Westerbom, Markku Viitasalo, Alf Norkko

**Affiliations:** ^1^ International Council for the Exploration of the Sea (ICES) DK‐1553 Copenhagen V Denmark; ^2^ Tvärminne Zoological Station University of Helsinki FI‐10900 Hanko Finland; ^3^ Biodiversity Natural Environment Centre Finnish Environment Institute (SYKE) P.O. Box 413 FI‐90014 Oulu Finland; ^4^ Metsähallitus Natural Heritage Services P.O. Box 94 FI‐01301 Vantaa Finland; ^5^ Marine Research Centre Finnish Environment Institute (SYKE) FI‐00251 Helsinki Finland

**Keywords:** Baltic Sea, ecological gradients, marine, nestedness, turnover

## Abstract

The majority of studies in metacommunity ecology have focused on systems other than marine benthic ecosystems, thereby providing an impetus to broaden the focus of metacommunity research to comprise marine systems. These systems are more open than many other systems and may thus exhibit relatively less discrete patterns in community structure across space. Metacommunity structure of soft‐sediment benthic invertebrates was examined using a fine‐grained (285 sites) data set collected during one summer across a large spatial extent (1700 km^2^). We applied the elements of metacommunity structure (EMS) approach, allowing multiple hypothesis of variation in community structure to be tested. We demonstrated several patterns associated with environmental variation and associated processes that could simultaneously assemble species to occur at the sites. A quasi‐Clementsian pattern was observed frequently, suggesting interdependent ecological relationships among species or similar response to an underlying environmental gradient across sites. A quasi‐nested clumped species loss pattern was also observed, which suggests nested habitat specialization. Species richness declined with depth (from 0.5 to 44.8 m). We argue that sensitive species may survive in shallower water, which are more stable with regard to oxygen conditions and present greater habitat complexity, in contrast to deeper waters, which may experience periodic disturbance due to hypoxia. Future studies should better integrate disturbance in terms of temporal dynamics and dispersal rates in the EMS approach. We highlight that shallow water sites may act as sources of recruitment to deeper water sites that are relatively more prone to periodic disturbances due to hypoxia. However, these shallow sites are not currently monitored and should be better prioritized in future conservation strategies in marine systems.

## Introduction

The metacommunity concept (Leibold et al. 2004) considers both local and regional processes in the context of the spatial organization of biological communities. A metacommunity can broadly be defined as a set of communities that are potentially interlinked by dispersal, whereas a community comprises the species occurring at an individual site (Holyoak et al. 2005). Two complementary approaches have been used to evaluate patterns of spatial variation within the metacommunity framework: a mechanistic model‐based approach (Cottenie 2005) and a pattern‐based approach (Leibold and Mikkelson 2002). The mechanistic approach focuses on spatially mediated models (i.e., patch dynamics, species sorting, mass effects, and neutral model) and their underlying mechanisms (e.g., dispersal, biotic interactions, or responses to abiotic environmental characteristics). In contrast, the pattern‐based approach of “elements of metacommunity structure” (EMS; Leibold and Mikkelson 2002) focuses on the distribution of multiple species along latent environmental gradients to identify best‐fit patterns that are related to the nonrandom species associations within the metacommunity (i.e., checkerboard, nested, evenly spaced, Gleasonian, or Clementsian patterns; sensu Leibold and Mikkelson 2002).

Analytical approaches searching for large‐scale patterns can provide much‐needed generality to small‐scale experimental approaches looking at biotic interactions and other community assembly mechanisms (Ricklefs 2008). By investigating the distribution of species rather than solely looking at the mechanisms determining species composition at a site (i.e., facilitation and competition), several nonrandom patterns can be identified and compared. Even though gradient studies have demonstrated turnover in community composition (Hoagland and Collins 1997), the EMS approach can show to what extent species composition changes when moving across gradients, or if species‐poor sites within the region represent a subset of species‐rich sites (Leibold and Mikkelson 2002). However, attempts to search for metacommunity patterns have often been performed in isolation (i.e., contrasting single idealized models with randomness, e.g., Haudsorf and Hennig 2007). Such an approach is liable to wrongly concluding that a metacommunity is randomly structured, as other potential distributional patterns have not been considering in the same analysis (Henriques‐Silva et al. 2013; Dallas and Presley 2014; Heino and Alahuhta 2015).

A subject of long‐lasting debate has been to what extent species ranges end at the same position or if species replace each other more or less continuously (Whittaker 1975). Clements (1961) first regarded communities as discrete entities. In contrast, Gleason (1926) described a pattern of continual change in species composition along environmental gradients without the formation of discrete assemblages, which result from idiosyncratic, species‐specific responses to the environment. Another pattern not directly considered by Clements (1961) or Gleason (1926) is nestedness (Patterson and Atmar 1986). It has been proposed that a nested pattern can emerge if species‐poor sites form nested subsets of increasingly species‐rich sites (Patterson and Atmar 1986). Nestedness can, however, be measured using various different indices, and the one we used in the context of the EMS may not be directly comparable to those used in many nestedness studies (see also Ulrich et al. 2007; Baselga 2010). In general, nestedness is attributed to either variation in habitat complexity or habitat quality between sites (Hylander et al. 2005), but it may also depend on species‐specific characteristics, such as dispersal ability, habitat specialization, tolerance to abiotic conditions (Heino et al. 2009). However, if strong interspecific competition exists between species, trade‐offs in competitive ability may manifest as distributions that are more evenly spaced along environmental gradients than expected by chance (Tilman 1982). Finally, if pairs of species co‐occur less than expected by chance (i.e., more‐or‐less mutually exclusive distributions) and if such pairs occur independently of other pairs, then a checkerboard pattern can be expected (Diamond 1975).

**Figure 1:**
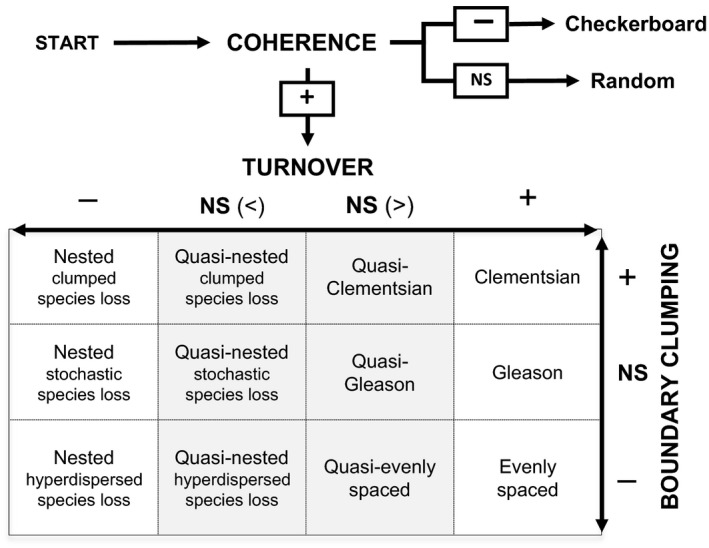
Analytical approach in the EMS analysis testing for coherence in metacommunity structure, and 12 best‐fit patterns for species distribution when testing for turnover and boundary clumping in metacommunities with significantly positive coherence. Significant positive results, +; significant negative results, −; nonsignificant, NS; fewer replacements than in random runs, (<); more replacements than in random runs (>). Quasi structures are shaded. Adapted from Presley and Willig (2009).

Using a stepwise procedure, the “elements of metacommunity structure” (EMS, see Fig. 1B) approach can simultaneously test for multiple idealized patterns across a set of sites (Leibold and Mikkelson 2002). Objective criteria based on coherence, turnover, and boundary clumping are used to assess the correspondence of an empirical data set with each of the hypothetical idealizations of species distribution (i.e., checkerboard, nested, evenly spaced, Gleasonian, or Clementsian patterns). This approach was first proposed by Leibold and Mikkelson (2002). Thereafter, the approach was refined by Presley et al. (2010) and has been considered an initial first step to applying metacommunity ecology to examine spatial organization of communities (Presley and Willig 2009). By comparing systems that differ in taxa and/or spatial extent, this approach may prove a useful tool in the search for general rules determining metacommunity structure. Empirical testing of the EMS approach has been mainly in terrestrial systems (Presley and Willig 2009; Presley et al. 2012; Meynard et al. 2013; Dallas and Presley 2014), for which several patterns of community structure have been demonstrated. Aquatic systems have largely been neglected when applying the EMS approach and, to our knowledge, the existing studies are from fresh waters (Henriques‐Silva et al. 2013; Dallas and Drake 2014; Erős et al. 2014; Fernandes et al. 2014; Heino et al. 2015). The generality of idealized patterns in metacommunity structure is yet without testing in marine systems. In marine systems, nonisolated communities are embedded across a continuum of environmental gradients in a highly connected system where dispersal may strongly influence community composition across multiple spatial and temporal scales (Whitlatch et al. 1998; Grantham et al. 2003; Cornell and Harrison 2013; Pilditch et al. 2015).

It can be expected that marine species will rarely respond to an identical set of environmental characteristics, although due to high rates of dispersal in a relatively open systems, it can be expected that communities will also share a suite of common species across sites (i.e., due to source–sink dynamics; Pulliam 1988). Species may thus experience change in abiotic characteristics as gradual or as a more‐or‐less discrete boundary. Nestedness, for example, has been reported in pelagic fish, for which depth was found to correlate with a steady decrease in species richness (e.g., Smith and Brown 2002). Nestedness can, however, be measured using various different indices, and the one we used in the context of the EMS may not be directly comparable to those used in many nestedness studies (see also Ulrich et al. 2007; Baselga 2010). The formation of discrete community types has also been observed at the local scale by investigating facilitation and mutualism using manipulative field experiments (e.g., Norkko et al. 2006). Interspecific and intraspecific competition has been widely reported in marine systems, for example, between barnacles (Connell 1961) or between filter‐feeding bivalves (Peterson and Andre 1980), and this knowledge has advanced ecological understanding of within‐community interactions (Menge and Sutherland 1976; Wilson 1991). It is unclear, however, to what extent spatial patterns across larger regions reflect these expected patterns in the degree of species co‐occurrence (Puri et al. 2013). Given the relatively more open nature of these systems, idealized patterns in marine metacommunity structure may occur less frequently. However, by applying the EMS approach, one can explore potential mechanisms operating at regional and local scales, thereby complementing small‐scale experimental approaches that examine the structuring mechanisms. Using the EMS approach in marine metacommunities may thus provide insight into the generality of several nonrandom patterns in the spatial organization of metacommunities. For example, in the nontidal Baltic Sea, variation in depth and salinity in coastal areas can occur in a relatively small geographic area and thus present a useful platform with which to address issues of metacommunity structure of soft‐sediment benthic invertebrates.

In this study, we examined whether coastal soft‐sediment benthic fauna exhibits any of the idealized metacommunity structures at a large scale comprising all sites, as well as within three smaller areas (Fig. 1A). We examined how latent environmental gradients in each of the EMS analysis are associated with species richness and total abundance, as well as measured environmental variables (i.e., salinity and depth) across sites. Thus, distinct mechanism(s) can better be associated with metacommunity structure within respective geographical groupings of sites.

Environmental heterogeneity in marine systems has been shown to vary depending on the spatial extent of a coastline considered (Connell and Irving 2008). This can especially be the case when considering a coastline with an extensive archipelago, supporting a variety of habitat types (e.g., rocky shores, soft‐sediment bays and lagoons). In our study area, we thus predict that the underlying environmental gradients may be more variable at a larger spatial extent, and thus, the metacommunity is more likely to exhibit positive turnover. In contrast, we predict negative turnover (i.e., nestedness) to be more likely at small spatial scales because dispersal will be less limiting and because environmental variable ranges are narrower with relatively lower limiting environmental gradient extremes. We thus predict that metacommunity structure will differ in response to either an increase or decrease in the spatial scale considered, as species will respond to space due to changes in environmental conditions or to a change in dispersal distance among sites. We also predict that both depth and salinity will correlate with gradients along which patterns of metacommunity structure are exhibited. In the coastal brackish water system of the study, species may have contrasting requirements depending on whether they are limited by either lower or higher salinity extremes. We therefore predict that discrete community types will replace each other along a salinity gradient, given the either hyperosmotic or hypo‐osmotic conditions that marine and freshwater species may experience in brackish water systems. Such a more‐or‐less abrupt change in community structure is best described by a Clementsian structure, rather than Gleasonian structure. In contrast, an increase in depth may occur across relatively smaller spatial scales, and we thus expect a decline in species richness as conditions for benthic invertebrates gradually become more constraining. We thus predict a nested metacommunity structure along such short gradients.

## Materials and Methods

### Study area

**Figure 2:**
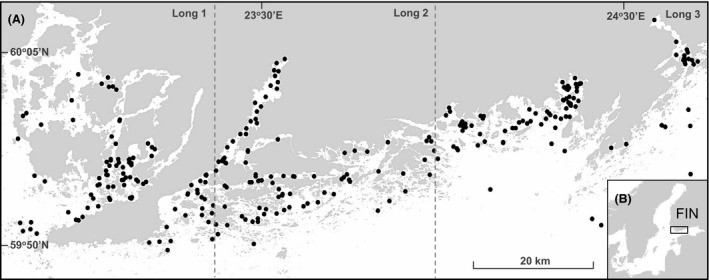
(A) Geographic position of study sites (solid black dots, *n* = 285) across a complex archipelago area in the northern Gulf of Finland. Hashed gray lines indicate finer spatial extent longitudinal groupings Long 1–3 of sites from west to east, respectively. (B) The insert shows the location of the study area (rectangle) within the Baltic Sea region along the coastal of Finland (FIN).

Our study area was located in the Gulf of Finland (Fig. 2), which has a gradient of decreasing salinity in an eastwards direction. The study region's coastline is composed of a complex archipelago, and thus, sites vary in the degree of openness to wind‐induced wave energy from a dominant southwest direction (Soomere et al. 2008; Fig. 2). The system has no regular tides (Soomere et al. 2008). Deeper water basins in the coastal archipelago areas are also prone to periodic disturbances (seasonal hypoxia, Bonsdorff et al. 1997). Across a gradient of inner‐to‐outer archipelago, sites located closer to the mainland have lower salinity due to freshwater run‐off and are more shallow and sheltered from wind‐induced waves. Exposure to wind and depth also determines sediment granulometry at a site, which may vary from silt to coarse gravel (Le Hir et al. 2007).

### Study organisms

In the study areas, dominant taxa include the bivalve *Macoma balthica* and the polychaetes *Marenzelleria* spp. Benthic invertebrate communities in the northern Baltic Sea can be characterized as being low in species richness, but having high total abundances (Bonsdorff and Pearson 1999). Sediment grain size characteristics have been shown to correlate well with the occurrence and abundance of benthic invertebrates (Gray 1974). In this region up to 40% of shallow benthic invertebrate species are brooders and lack a larval dispersal phase (Valanko et al. 2010a). Frequent small‐scale dispersal is, however, common for soft‐sediment benthic invertebrates, as individuals are not permanently attached to the substrate. Postlarval dispersal rates are highest when wind‐induced waves exceed a long‐term average (Valanko 2012), which can be relatively more important in maintaining community composition (Valanko et al. 2015), and site‐to‐site variation in initial larval recruitment can be considered to be largely independent of local adult abundances (Pedersen et al. 2008; Pineda et al. 2009).

### Sampling and processing of invertebrates

A fine‐grained (285 local sites) soft‐sediment benthic community data set across a large spatial extent (1700 km^2^) was collected in late summer (August–September) 2012, when postsettled juveniles were relative large, and before the onset of autumn storms. Sampling stations were selected by random stratification, using modeled depth, wave exposure, salinity, and turbidity. The selection criteria of sites were designed to maximize the number of sites visited and, cover a maximally broad area, within a reasonably short sampling period. Samples were collected using a PONAR grab sampler (12.5 × 12.5 cm). Samples were sieved using a 0.5‐mm mesh and preserved in 70% ethanol. Samples were sorted and enumerated using a binocular microscope to the lowest practical level, using available identification keys. Benthic community composition in replicate samples was characterized by a low number of taxa and high abundances. The chosen scale (12.5 × 12.5 cm) was considered a sufficiently small enough scale at which species interactions occur, and we have thus defined one replicate to comprise our measure of a local community in this study. Within the context of analyses of EMS, a metacommunity is defined as a set of ecological communities at different sites (potentially but not necessarily linked by dispersal), whereas a community is a group of species at a given site (Leibold and Mikkelson 2002). Thus, two scales are assumed in this metacommunity study: local (a site) and regional (the study region). At the local scale of a site, local‐scale processes such as site‐specific environmental conditions and species interactions are expected to affect population dynamics up to the point of local extinctions. Patterns detected may also be affected by regional processes (e.g., dispersal between sites) occurring across local sites. It is, however, worth noting that in continuous marine systems, local and regional scales are more likely to merge than in other less well‐connected systems, such as a set of lakes.

### Measured environmental variables

GPS coordinates (decimal degrees) and depth data were measured at sites at the time of sampling, while salinity was interpolated using a spline method in ArcGIS 9.2 on a 25 m × 25 m raster based on measurements taken in the mid‐summer period (1.7–31.8) over the years 1999–2008 (Finnish Environment Institute). Validation of interpolated salinity was done using a smaller data set of 72 sites (salinity range 0.8–6.2, S. Valanko et al., unpubl. data), for which a strong correlation was observed (*r* ^2^ = 0.96, *P *<* *0.001).

### Data analysis

Data were analyzed as a whole (*n* = 285), as well as within three study areas along the coast (Long 1 = 22°49.673–23°22.274, Long 2 = 23°22.412–23°58.272, Long 3 = 23°58.939°24°41.242, see Fig. 1A). This was done to explore whether environmental variation within smaller subregions would result in different patterns in metacommunity structure. A Levene's test was performed to see how much measured salinity and depth of sites varied between groups (Long 1–3). This test calculates for each group (*n* = 95) its average distance to an overall centroid value of salinity or depth of sites (*n* = 285) and then performs a test to see whether the three group's distance to group centroid differ significantly from each other with respect to that variable. We also examined environmental heterogeneity within each subset of sites to guarantee ecological basis in our comparisons of the subsets of sites.

### Elements of metacommunity structure (EMS)

In contrast to ordering sites along a specifically measured environmental variable, the EMS analysis allows the metacommunity itself to define the gradient(s) of response (Leibold and Mikkelson 2002). First, a site‐by‐species incidence matrix was constructed separately for all data sets (i.e., all sites and longitude 1–3, Fig. 1A). Matrices were then ordered using both the primary (axis 1) and secondary (axis 2) axis extracted via reciprocal averaging (i.e., correspondence analysis), which optimizes the proximity of species with similar distributions and the proximity of sites with similar species composition (Gauch 1982; Legendre and Legendre 2012). In so doing, it allows the composition of communities and occurrence of species to define the gradient that is most important to metacommunity structure.

Based on the ordinated site‐by‐species incidence matrix, coherence, species range turnover, and boundary range clumping were determined (Fig. 1B) using both primary and secondary axes within each site grouping separately. Coherence tests whether species are responding to the same gradient by calculating the number of embedded absences within species ranges (Leibold and Mikkelson 2002). Negative coherence (i.e., more embedded absences than expected by chance) suggests a checkerboard pattern where species occurrences are more‐or‐less mutually exclusive of one another, while positive coherence (i.e., less embedded absences than expected) suggests that occurrences and absences of species are reacting to the same latent environmental gradient and are not scattered along a gradient (i.e., random). For metacommunities exhibiting positive coherence, 12 possible nonrandom structures can be identified by testing for different combinations of turnover and boundary clumping (Presley and Willig 2009; Fig. 1). First, turnover is tested for by looking at whether species ranges are nested within each other or whether they are replacing each other when moving across the gradient (Leibold and Mikkelson 2002). Thus, the number of replacements can either be significantly or nonsignificantly (quasi structures) greater or less than expected. Second, boundary clumping is determined, which tests how often multiple species have their range limits at the same site across the gradient. Boundary clumping is tested using Morisita's I index, which can be clumped (positive, I > 1), stochastic (nonsignificant, NS), or hyperdispersed (negative, I < 1).

The significance of the index value for coherence and turnover was tested using a fixed‐proportional null model, which maintains species richness of each site (i.e., row sums are fixed), but species ranges are filled based on their marginal probabilities (i.e., the “r1” null model; Dallas 2014; Gotelli 2000). We used 1000 simulations to provide random matrices. Index values derived from randomization were then compared to the observed index values to assess statistical significance.

We interpreted the results of the EMS analysis according to Presley et al. (2010) and used the *metacommunity* function in the metacom package for calculations (Dallas 2014) in the R environment (version 3.0.1, R Core Team 2013). In addition, Spearman rank correlation was used to test whether latent environmental gradients (i.e., primary and secondary axis site scores from correspondence analysis) were significantly correlated with measured environmental parameters (i.e., longitude, salinity, and depth), as well as with species richness and total abundance (Presley et al. 2009, 2010; Meynard et al. 2013).

## Results

A total of 38 species and 18,879 individuals were recorded at the 285 sites across the study region. Number of species at sites varied between 1 and 17, while abundance varied from 1 to 1056 individuals per grab. On average, 6.0 (SE: 0.2) species and 66.3 (SE: 5.7) individuals were found per site. The species present at many sites also had higher overall abundance, that is, the occupancy–abundance relationship was very strong (*R* ^2^ = 0.77, *P *<* *0.001).

Across all 285 sites, the EMS analysis revealed a random pattern across CA axis 1 and positive coherence with a quasi‐Clementsian gradient along CA axis 2 (Table 1). Correspondence analysis eigenvalues of the secondary CA axis (0.294) were only slightly smaller than that of the primary CA axis (0.338), accounting, respectively, for 6.3% and 7.2% of variability of total inertia. The quasi‐Clementsian pattern across CA axis 2 was also associated with a significant change in both salinity and depth. Salinity was positively correlated and depth negatively correlated with CA axis 2. Species richness and total abundance were also significantly correlated with CA axis 2, while none of these variables was significantly related to CA axis 1. Closer examination of metacommunity structure at a finer spatial extent within longitudinal ranges (Long 1–3) also revealed positive coherence on both their primary or secondary CA axis (Table 1). These site groupings were best described by either a quasi‐Clementsian pattern or a quasi‐nested pattern with clumped species loss. In general, site scores on the primary and secondary CA axis within different groupings exhibited significant but weak correlation with salinity and depth (Table 2).

**Table 1:** EMS analysis conducted for soft‐sediment benthic invertebrate metacommunity, for all sites (*n* = 285) and finer spatial extent longitudinal groupings (Long 1–3, each *n* = 95). Coherence: the number of embedded absences (Abs) significance (*P*), relative to a simulated null matrix (Mean) and its standard deviation (SD). Bold denotes significant coherence (<0.05), a prerequisite to consider turnover and boundary clumping. Turnover: the number of species replacements (Repl) its significance (*P*) relative to simulated null matrices (Mean) and its standard deviation (SD). Boundary clumping: based on the Morisita's index (index) and its significance using a chi‐squared test

	Coherence					Turnover					Boundary clumping			Metacommunity pattern	df
	Abs	*P*	Mean	SD		Repl	*P*	Mean	SD		Morisita's index	*P*			
CA 1															
All sites	5640	0.198	6216	447.6		765412	0.381	605739	182100.7		4.697	0.000		Random	37
Long 1	**1132**	**0.000**	1628	116.2	+	121682	0.062	78939	22920.6	**+**	2.580	0.000	>1	Quasi‐Clementsian	31
Long 2	**1037**	**0.000**	1577	148.2	+	96276	0.334	74780	22231.7	**+**	2.556	0.000	>1	Quasi‐Clementsian	32
Long 3	1015	0.118	1196	115.7		53597	0.301	40444	12711.5		3.850	0.000		Random	25
CA 2															
All sites	**4593**	**0.000**	6249	467.4	+	758403	0.372	595119	182932.0	**+**	2.910	0.000	>1	Quasi‐Clementsian	37
Long 1	**1358**	**0.014**	1634	112.5	+	80987	0.859	76944	22837.5	**+**	4.234	0.000	>1	Quasi‐Clementsian	31
Long 2	**1039**	**0.000**	1572	148.1	+	74807	0.974	75557	23112.9	−	3.304	0.000	>1	Quasi‐nested clumped species loss	32
Long 3	1021	0.129	1197	116.0		50966	0.365	39886	12221.6		2.430	0.000		Random	25

**Table 2:** Spearman rank correlation (*ρ*), corresponding *P*‐value, and significance (<0.05) in bold for association for all sites (*n* = 285) and finer spatial extent longitudinal groupings (Long 1–3, each *n* = 95) salinity, depth, species richness, total abundance, and site scores for primary and secondary CA axis extracted via reciprocal averaging

	Salinity		Depth		Species richness		Total abundance	
	*ρ*	*P*‐value	*ρ*	*P*‐value	*ρ*	*P*‐value	*ρ*	*P*‐value
CA 1								
All sites	**0.191**	**0.001**	**0.164**	**0.006**	**0.238**	**0.000**	**0.299**	**0.000**
Long 1	**0.292**	**0.004**	**−0.330**	**0.001**	**0.487**	**0.000**	**0.458**	**0.000**
Long 2	**0.398**	**0.000**	0.054	0.600	**0.223**	**0.030**	0.116	0.262
Long 3	0.166	0.107	**0.446**	**0.000**	**−0.345**	**0.000**	−0.067	0.519
CA 2								
All sites	−**0.243**	**0.000**	**0.280**	**0.000**	**−0.502**	**0.000**	**−0.325**	**0.000**
Long 1	−0.097	0.350	**0.619**	**0.000**	**−0.508**	**0.000**	−0.085	0.415
Long 2	−**0.252**	**0.014**	0.149	0.149	**−0.369**	**0.000**	−**0.246**	**0.017**
Long 3	0.146	0.159	**0.204**	**0.048**	**0.504**	**0.000**	**0.496**	**0.000**

Sites were grouped into three areas within longitudinal ranges (Long 1–3), which enabled us to examine metacommunity structure between regions at a finer spatial extents than the whole study area. The most westerly grouping of sites (Long 1) exhibited a quasi‐Clementsian gradient across the first and second CA axes, while the central longitudinal grouping of sites (Long 2) displayed both a quasi‐Clementsian and a nested clumped species loss pattern on the first and second CA axis, respectively. The Long 2 site grouping was also significantly more variable in salinity (1.6–5.7) than in other longitudinal site groupings (*F* = 130.6, *P *<* *0.001, Table 3), while Long 1 site grouping exhibited significantly (*F* = 5.0, *P *=* *0.030) larger variation in depth (1.1–44.8 m). The most easterly longitudinal range (Long 3) did not exhibit significant coherence in metacommunity structure (i.e., they showed randomness). Long 3 showed a larger longitudinal range (*F*: 6.8, *P *=* *0.003, Table 3) and exhibited relatively less variation in salinity (4.6–5.6) than the other two longitudinal site groupings. Long 3 grouping of sites also had a lower species pool (26 taxa) than the other two longitudinal groupings of sites (Long 1 and Long 2).

**Table 3:** Levene's test comparing variation in salinity and depth separately within finer spatial extent longitudinal groupings (Long 1–3, each *n* = 95). (*F*) strength, (*P* perm) significance with <0.05 denoted in bold, (Mean) distance to group centroid, (SE) standard error of estimate

Salinity					Depth					df1	df2	Size
PERMDISP		Deviation fom centroid			PERMDISP		Deviation fom centroid					
*F*	(*P*)perm	Group	Mean	SE	*F*	(*P*)perm	Group	Mean	SE			
**130.6**	**0.001**	Long 1	0.28	0.02	**5.0**	**0.030**	Long 1	7.1	0.6	2	282	95
		Long 2	1.06	0.07			Long 2	4.7	0.4			95
		Long 3	0.15	0.01			Long 3	6.1	0.6			95

## Discussion

Species may experience environmental gradients as gradual or as more‐or‐less discrete boundaries, depending on species‐specific characteristics (e.g., dispersal ability, habitat specialization, tolerance to abiotic conditions; Presley et al. 2011). When examining patterns at a large spatial extent across all study sites, we found significant coherence in metacommunity structure. In addition, across the latent environmental gradient (measured as CA axis 1 and 2), species richness and total abundance increased, concomitant to increasing salinity and decreasing depth. This finding suggests that species occurrence is determined by responses to underlying environmental gradients in the study region. However, the number of replacements was not significantly greater than the randomly generated null model pattern, whereas boundary clumping was positive and significant. Such a quasi‐Clementsian pattern is presumed to be characteristic of metacommunities, where the majority of species span a large portion of the latent environmental gradient with a clumped Clementsian structure at the end of the gradient (Presley et al. 2010, 2012). A Clementsian structure suggests either interdependent ecological relationships among species or a similar response to underlying environmental thresholds across sites in the study area (i.e., salinity and/or depth).

In our study, we defined a site to be the appropriate scale for the population dynamics that underlie the mechanisms invoked to explain the patterns of EMS. Given the fact that species may vary in the size of their individual “local” populations, it is likely that local and regional scales may merge for different species at different spatial extents. We, however, believe that the patterns discovered in this study represent real biological gradients across the 1700 km^2^ study area, and do not represent error in estimating the scale of a community. Across larger spatial extents, there is more room for variation in environmental conditions, and thus, greater differences in species composition between sites (i.e., species turnover) can be expected due to niche differentiation (Presley et al. 2010). In addition to underlying environmental gradients, dispersal limitation is more likely at a larger spatial extent or then species may have interdependent ecological relationships. These potential mechanisms may result in a threshold‐like response in metacommunity structure (expectation: Clementsian). We initially predicted that lower salinity sites in the inner archipelago areas in the north would favor species with a freshwater origin, while higher salinity sites in the outer archipelago in the south would favor species with more a marine origin (Bonsdorff 2006; Zettler et al. 2013). Across such a sharp boundary, it can be expected that communities exhibit high turnover in species composition between sites and, hence, that there are groups of species which respond in the same manner to the environment (expectation: Clementsian). Even though significant, salinity was not strongly associated with the latent environmental gradients across sites which had a quasi‐Clementsian structure. It is thus important to consider the predictive power of modeled salinity or other structuring mechanisms (not considered in this study) that may be driving turnover in species composition across sites. Despite the large number of sites, our study region as a whole was also limited in spatial extent with regard to fully marine and fully freshwater extremes of the entire Baltic Sea region. It is interesting to speculate whether metacommunity patterns would tend more toward a t rue Clementsian structure, associated with salinity, if the study region had extended across the entire Baltic Sea (e.g., Villnäs and Norkko 2011; Zettler et al. 2013).

**Figure 3:**
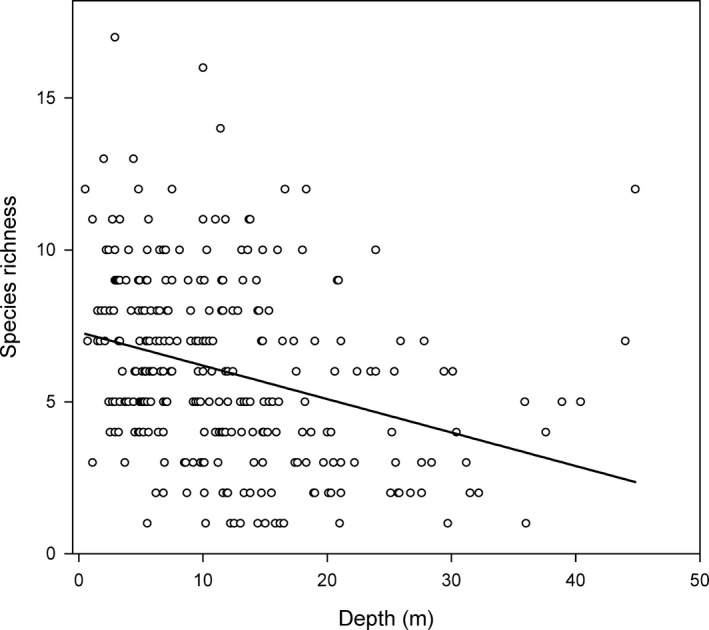
Pearson's r correlation (solid line, −0.307, *P *<* *0.001) between species richness and depth across study sites (*n* = 285).

In addition to quasi‐Clementsian structure, nonsignificant coherence in metacommunity structure (i.e., randomness) was observed. Species richness was also found to decrease toward deeper water sites (all sites = −0.307, *P *<* *0.001, Fig. 3), which is in agreement to previous studies in the region (e.g., Bonsdorff 2006). These observations make it interesting to speculate whether species are assembling less frequently at deeper water sites that are prone to disturbance from periodic hypoxia, thus increasing the number of embedded absences (i.e., randomness) in species range toward deeper water sites. Even though we did not measure oxygen conditions directly, in our study region, increasingly larger and more frequent benthic disturbances have also been reported in both coastal and offshore areas of the Baltic Sea due to hypoxia (Bonsdorff et al. 1997; Conley et al. 2011). In a complex archipelago setting, such as our study region, there will also be variation in bottom topography, exposure, and water exchange characteristics across a relatively smaller spatial extent (Bonsdorff et al. 1997). Oxygen conditions will also depend on temporal variation seasonally (e.g., temperature, ice‐cover, peaks in primary productivity) and interannually (e.g., oxygen, salinity). A threshold of hypoxic stress exists (O_2_ < 2 mg L^−1^) beyond which even the most common species cannot survive for a prolonged period of time (Conley et al. 2011; Villnäs et al. 2012), such as the bivalve *Macoma balthica* (present at 83.2% of sites) or the polychaetes *Marenzelleria* spp. (present at 67.0% of sites). Sites with conditions around this threshold may contain only random assemblages of transient species rather than a subset of species characteristic of that part of an environmental gradient. Disturbance can also produce species‐abundance distributions that are strongly dominated by one or two species (Bloch et al. 2007). Benthic communities in our study were characterized by relatively low species richness but high overall abundances (mean: 530 individual per m^2^, SE: 45.6, max: 8448 individuals per m^2^). With regard to oxygen conditions, vulnerable species may thus survive in shallower water that are more stable and present greater habitat complexity in sediment grain size characteristics. Deeper sites can thus be expected to be subject to longer periods of oxygen depletion and may thus increase the number of embedded absences in species ranges (i.e., randomness) in metacommunity structure. Within this context, assembly history and dispersal between sites may also be important mechanisms contributing to realized patterns (or lack of) in metacommunity structures between sites (see also Presley et al. 2010). Similarly, it has been suggested that changes in community structure are likely to be profound at sites that periodically experience large‐scale disturbances (sensu White and Pickett 1985), so that a community is least structured or, alternatively, most random, immediately following disturbance.

Multiple factors may be acting simultaneously to assemble species across sites, including environmental factors and dispersal, and those factors may vary temporally (Erős et al. 2014; Fernandes et al. 2014). Benthic environments are dynamic in space and time, varying in the degree of harshness exerted on invertebrate species inhabiting them. However, the analysis of metacommunity structure based on site‐by‐species incidence matrix used in this study cannot detect the effects of dispersal even if they exist. It can be assumed that species will only assemble at a site if dispersal has been sufficient and if abiotic environmental conditions of a site match their requirements (Chase and Leibold 2003; Cottenie 2005). Dispersal limitation between sites may thus prevent community structure to recover from the effects of a stressor (Heino 2013; Pilditch et al. 2015). Furthermore, continued dispersal of individuals beyond a species' optimal range limits can also create presences of species at suboptimal sites due to high dispersal rates from environmentally suitable sites (Leibold et al. 2004). As opposed to “perfect” metacommunity structure, marine systems are subject to periodic disturbance and are relatively more open (Whitlatch et al. 1998; Grantham et al. 2003; Valanko et al. 2010b), which may impede species from tracking their idealized niches or from forming idealized interdependent relationships with other species. In open systems, both a surplus of dispersal (i.e., mass effects) and dispersal limitation (i.e., related to disturbance history) may be important structuring mechanisms for the observed metacommunity structure. In addition, in other connected and dynamic systems, such as streams and floodplain lakes, the best‐fit patterns of metacommunity structure may vary in time (Erős et al. 2014; Fernandes et al. 2014; Cisneros et al. 2015). Hence, we can expect that different metacommunity structures might emerge in our study system when sampled in different times of the year or in different years.

Upon closer investigation of finer spatial extent areas in addition to also finding quasi‐Clementsian structure, a quasi‐nested pattern with clumped species loss in metacommunity structure was also observed. These two structures represent almost opposite ends when testing for the number of replacement (i.e., significantly more or less). The extent to which species turnover occurs between sites under consideration should depend on the spatial scales under investigation relative to dispersal limitation, as well as the overall variations in environmental conditions within the study area. It can thus be challenging to assign one particular mechanism to explain nested patterns along gradients if an obvious environmental variable is not correlated with species richness (Presley and Willig 2009). However, in our study, variation in species richness of Long 2 site groupings with a quasi‐nested clumped species loss was significantly correlated with depth (Pearson's *r*; −0.509, *P *<* *0.001). One can thus expect a predictable pattern of species loss at finer spatial extents with an increase in depth, with species that are absent from a particular site, also being absent from all sites with fewer species (Presley and Willig 2009). However, much like elevation in terrestrial systems (Whittaker 1956; Presley et al. 2012), it is likely that depth is acting as a surrogate measure of some other variables that better relate to benthic habitat characteristics. A particular strength of the EMS approach is that it can bring further ecological understanding of how species loss occurs by testing boundary clumping (Presley and Willig 2009). In our study, nested pattern indicated significant positive boundary clumping (Morita's I index > 1, Table 1), which has been suggested to be characteristic of a situation when habitat specialization determines the location of species range boundaries (i.e., ecotones). A distinct clumped pattern of species loss has, for example, been demonstrated in bats across an elevational gradient in the eastern Andes, which was associated with changes in habitat type along the elevational gradient (Presley et al. 2010, 2012). In marine systems environmental characteristics (e.g., oxygen, temperature, salinity, light, wave attenuation, sediment characteristics) to which species respond change with depth in a predictable fashion across small spatial extents. Depending on water turbidity, light penetration decreases with depth. Shallow water will thus have higher productivity, while deeper water is relatively cooler (thermocline) and also have higher salinity (halocline). This stratification also limits mixing of oxygen and nutrients between deeper and shallower water. Wave energy reaches the seafloor more often in shallow water. As a result, shallow water sites exposed to waves have greater rates of sediment erosion, transport and deposition. Sediment characteristics can thus tend toward coarse gravel at shallow sites that are exposed to waves and currents, while deeper and/or more sheltered sites will tend toward finer mud (Gray 1974; Le Hir et al. 2007). It is thus likely that our results reflect the fact that, across relatively finer spatial extent (i.e., shorter dispersal distances) and subsequent changes in environmental conditions associated with depth, a nested community structure may be a result of species responding to both the larger variation in habitat types in shallow water and to the gradually limiting environmental conditions in deeper waters. This reasoning is suggested by the significant, but weak, correlations of depth along the latent environmental gradient that exhibited a quasi‐nested with clumped species loss.

### Implications for conservation and monitoring

Increased ecological understanding of the spatial organization of communities can also help identify priorities for conservation efforts to curb effects of anthropogenic stressors (e.g., eutrophication in the Baltic Sea). For example, Hylander et al. (2005) have suggested that by differentiating between nestedness that arises due to habitat quality or nested habitats, conservation efforts can better be targeted at either species hotspots or sites with diverse habitats. Our study suggests a nested clumped species loss pattern. Thus, in contrast to only prioritizing species richness hotspots (i.e., nestedness due to habitat quality), our findings also suggest that shallow sites in which representative habitat types (i.e., in terms of sediment grain size characteristics) are well developed should be a priority in future coastal conservation strategies. Coastal soft‐sediment benthic systems have been identified as critical habitats for many species, linking the sea with land and freshwater habitats (Levin et al. 2001; Cowen and Sponaugle 2009). Furthermore, frequent small‐scale dispersal in shallow soft‐sediments is common (e.g., Pilditch et al. 2015; Valanko et al. 2015) and may thus act to enhance species richness between shallow water sites, as well as to deeper water sites that are more prone to periodic disturbances. An important consideration is that, despite their potential importance, shallow site data considered in this study has rarely been gathered, mainly due to difficulty in accessing such sites by larger research vessels. Our study suggests, however, a re‐evaluation of present monitoring program practices focusing almost solely on offshore areas.

## Conclusion

While factors governing the distribution patterns across large spatial extents are complex, we found several patterns in metacommunity structure associated with environmental variation across salinity and depth gradients. However, dispersal and temporal trends should be better incorporated in analysis of metacommunity structure, as conclusions based on single time periods may not characterize the dynamics of a study system. Further studies are thus warranted to better identify multiple environmental gradients and mechanisms underlying species distribution patterns and metacommunity dynamics in continuous marine systems open to dispersal between sites. We conclude that very complex metacommunity structures may be a feature of open systems with high connectivity between sites.

## Conflict of Interest

None declared.
